# Long-term predictive value of acute kidney injury classification in diffuse proliferative lupus nephritis with acute kidney injury

**DOI:** 10.1186/s12882-019-1676-4

**Published:** 2020-01-13

**Authors:** Tianxin Chen, Ying Zhou, Jianna Zhang, Chaosheng Chen, Jingye Pan

**Affiliations:** 1 Department of Nephrology, The First Affiliated Hospital of Wenzhou Medical University, Wenzhou, 325000 Zhejian Province People’s Republic of China; 2 Department of ICU, The First Affiliated Hospital of Wenzhou Medical University, Wenzhou, 325000 Zhejiang Province People’s Republic of China

**Keywords:** Acute kidney injury, Lupus nephritis, KDIGO, ESRD, ACR;SLE

## Abstract

**Background:**

The long-term predictive ability of acute kidney injury (AKI) classification based on “Kidney Disease: Improving Global Outcomes”(KDIGO) AKI diagnosis criteria has not been clinically validated in diffuse proliferative lupus nephritis (DPLN) patients with AKI. Our objective was to assess the long-term predictive value of KDIGO AKI classification in DPLN patients with AKI.

**Methods:**

Retrospective cohort study was conducted by reviewing medical records of biopsy-proven DPLN patients with AKI from the First Affiliated Hospital of Wenzhou Medical University between Jan 1, 2000 and Dec 31, 2014. Multivariate Cox regression and survival analysis were performed.

**Results:**

One hundred sixty-seven DPLN patients were enrolled,82(49%) patients were normal renal function (No AKI), 40(24%) patients entered AKI-1 stage (AKI-1), 26(16%) patients entered AKI-2 stage (AKI-2) and 19(16%) patients entered AKI-3 stage (AKI-3). The mean follow-up of all patients was 5.1 ± 3.8 years. The patient survival without ESRD of all patients was 86% at 5 years and 79% at 10 years. The patient survival rate without ESRD at 10 yr was 94.5% for No AKI patients, 81.8% for AKI-1 patients, 44.9% for AKI-2 patients and 14.6% for AKI-3 patients. The area under the ROC curve for KDIGO AKI classification to predict the primary end point was 0.83 (95% CI: 0.73–0.93) (*P* < 0.001). In Cox regression analysis, AKI stage was independently associated with primary endpoint, with an adjusted hazard ratio (HR) of 3.8(95% CI 2.1–6.7, *P* < 0.001).

**Conclusion:**

Severity of AKI based on KDIGO AKI category was associated with progression to ESRD in DPLN patients. Analytical data also confirmed the good discriminative power of the KDIGO AKI classification system for predicting long-term prognosis of DPLN patients with AKI.

## Background

Lupus nephritis (LN) represents a common organ involvement in patients with systemic lupus erythematosus (SLE), and diffuse proliferative lupus nephritis (DPLN) remains a major cause of renal failure and mortality among patients with SLE [1–4]. Clinical reports found that complete or partial remission, nephritic flares and renal function at baseline were predictors of long-term renal outcome in LN [5–7]. Acute kidney injury (AKI) is a common complication of LN. 66 (20.5%) patients were identified as AKI among 322 Chinese LN patients in a recent report [8]. Fifty patients with acute renal failure (ARF) of 127 pediatric LN patients were identified in a prospective observational study [9]. Our previous study showed 54% DPLN patients had AKI and AKI severity was associated with an increase in renal function progression [10]. However, the long-term predictive ability of AKI classification based on “Kidney Disease: Improving Global Outcomes” (KDIGO) AKI diagnosis criteria has not been clinically validated in DPLN patients with AKI [11]. In recent years, the focus on the immediate complications and survival of AKI has been followed by a growing awareness of adverse long-term outcomes [12]. To assess the long-term predictive value of KDIGO AKI classification for patient and renal survival in DPLN patients, we now report the results of our extended follow-up of a large cohort of Chinese DPLN patients with AKI.

## Methods

This is a retrospective observational study. The Ethics Committee of the First Affiliated Hospital of Wenzhou Medical University waived the need for an informed consent. Data were collected anonymously.

### Participants

All biopsy-proven DPLN patients admitted to the First Affiliated Hospital of Wenzhou Medical University between Jan 1, 2000 and Dec 31, 2014 were enrolled retrospectively. The entry criteria: patients were diagnosed as SLE according to American College of Rheumatology (**ACR**) criteria [13] and had a histological diagnosis of DPLN (ISN/RPS classification: LN type IV or type IV + V) [14]. Patients with pre-renal AKI, post-renal AKI, renal tubular necrosis and acute interstitial nephritis; chronic renal insufficiency and obvious chronic histological changes were excluded. KDIGO AKI stages (Table 1) were classified according to serum creatinine (Scr) values on the first day of admission. One hundred sixty-seven DPLN patients were enrolled, 82 patients were normal renal function (No AKI), 40 patients entered AKI-1 stage (AKI-1), 26 patients entered AKI-2 stage (AKI-2) and 19 patients entered AKI-3 stage (AKI-3).

**Table 1:** Classification and staging of KDIGO criteria

Stage	KDIGO serum creatinine criteria
1	1.5 to 1.9 times baseline *or* ≥ 0.3 mg/dl (≥26.5 μmol/l) increase
2	2.0 to 2.9 times baseline
3	3.0 times baseline *or* increase in serum creatinine to ≥4.0 mg/dl (≥353.6 μmol/l) *or* initiation of renal replacement therapy *or* in patients < 18 years a decrease in eGFR to < 35 ml/minute per 1.73 m^2^

*Scr* Serum creatinine, *eGFR* estimated glomerular filtration rate

### Treatment protocol

Therapeutic decisions were taken in view of clinical conditions and the renal biopsy results. All patients initially received high dose prednisone (1 mg/kg per day) or intravenous methylprednisolone (MP) pulses (0.5–1.0 g/day for three consecutive days) followed by oral prednisone 0.5–1.0 mg/kg per day. After the initial 4 weeks of treatment, the dosage of prednisone was gradually tapered to 10-15 mg/day during 24 weeks in patients who improved clinically. Another course of intravenous MP pulses or the initial high-dosage prednisone for an additional 4 weeks were continued in patients with worsening renal function. Patients received intermittent intravenous cyclophosphamide (CTX) or mycophenolate mofetil (MMF) therapy. Maintenance therapy included low dose prednisone, hydroxychloroquine, and MMF.

### Data collection

Demographic an clinical records, renal pathological changes and laboratory test reports were retrieved. Demographic records included age, sex and duration of hospital stay. Clinical data included medical history, physical examination, diagnosis, medications and renal replacement therapy. Pathological data included microscopic and immunofluorescent characteristics. Laboratory data mainly included Scr, serum albumin, hemoglobin (Hb), 24-h proteinuria, anti-dsDNA antibody (Ab) and serum complement component 3(C3). Scr was used to calculate the estimated glomerular filtration rate (eGFR) assessed by the Chronic Kidney Disease Epidemiology research group (CKD-EPI) equation [15].

### Study end point and follow-up

The primary study end point was death and ESRD. ESRD was defined as maintenance dialysis (hemodialysis or peritoneal dialysis) more than 3 months or renal transplantation. Scr was measured at least once weekly in hospital. Follow-ups were carried out in outpatient service and Scr was measured monthly after patients discharged from hospital.

### Statistical methods

Values of continuous variables were reported as means ± standard deviation (SD), and compared by analysis of variance (ANOVA) test. Categorical data were reported as percentages and tested using the chi-square test. The survival analysis was based on the Kaplan-Meier curve with subjects censored for death and ESRD. A log-rank test was used to compare the survival rates among four groups. A multivariate Cox regression analysis was used to evaluate mortality and ESRD risk. Results were expressed as a hazard ratio (HR) with 95% confidence intervals (CIs). Discriminative power of AKI stage was assessed using the area under a receiver operating characteristic (AUROC) curve. All statistical tests were two-tailed; statistical significance was defined as *P* ≤ 0.05. Data were analyzed using the SPSS version 16 (SPSS, Inc., Chicago, IL, USA).

## Results

### Baseline clinical characteristics

One hundred sixty-seven DPLN patients were enrolled. 49% of patients were No AKI, 24% entered AKI-1,16% entered AKI-2 and 11% entered AKI-3 according to KDIGO AKI class criteria on the first day of admission. The baseline clinical characteristics on the basis of AKI stage are shown in Table 2. There were no significant differences in age, gender, anti-dsDNA Ab, C3 or cytotoxic agents treatment among four groups at baseline; however, the mean level of Scr, eGFR, blood pressure, proteinuria, serum albumin,and Hb at baseline was significantly different among four groups. The rate of MP pulse treatment was 24, 40, 62 and 68% in group No AKI, AKI-1, AKI-2 and AKI-3 (*P* < 0.01).

**Table 2:** Baseline clinical and serologic characteristics

Parameter	no AKI	AKI-1	AKI-2	AKI-3	*P*
n(%)	82 (49)	40 (24)	26 (16)	19 (11)	
Age (yr,mean ± SD)	29 ± 9	30 ± 10	32 ± 12	30 ± 12	0.44
Female(n[%])	76 (93)	39 (98)	23 (89)	17 (90)	0.49
systolic BP (mmHg;mean ± SD)	132 ± 23	141 ± 22	139 ± 24	150 ± 28	0.02
diastolic BP (mmHg;mean ± SD)	86 ± 16	91 ± 15	88 ± 16	98 ± 14	0.01
Scr (mg/dl;mean ± SD)	0.8 ± 0.1	1.4 ± 0.1	2.1 ± 0.4	5.9 ± 5.0	< 0.01
Serum Alb(g/dl;mean ± SD)	2.7 ± 0.7	2.5 ± 0.6	2.4 ± 0.6	2.3 ± 0.5	0.01
Hb(g/dl;mean ± SD)	10.2 ± 2.1	9.6 ± 2.1	8.8 ± 2.1	7.4 ± 1.7	< 0.01
Anti-dsDNA Ab positivity(n[%])	64 (78)	29 (73)	21 (81)	15 (79)	0.86
nephrotic syndrome(n[%])	55 (67)	33 (83)	23 (89)	17 (90)	0.03
C_3_(mg/dl;mean ± SD)	0.4 ± 0.2	0.4 ± 0.2	0.3 ± 0.2	0.4 ± 0.1	0.76
Proteinuria(g/d;mean ± SD)	3.7 ± 2.3	4.8 ± 3.0	4.5 ± 2.8	5.7 ± 4.7	0.03
eGFR (ml/min;mean ± SD)	92 ± 23	49 ± 6	32 ± 7	17 ± 18	< 0.01
MP pulse,(n[%])	20 (24)	16 (40)	16 (62)	13 (68)	< 0.01
MMF,(n[%])	34 (42)	15 (38)	11 (42)	6 (32)	0.85
CTX,(n[%])	58 (73)	26 (65)	20 (77)	11 (68)	0.47

*Scr* serum creatinine, *Alb* albumin, Hb hemoglobin, *Ab* antibody, *C3* complement component 3; *eGFR* mofetil, *CTX* cyclophosphamide

### Renal histological features

The histological features on the basis ISN/RPS are shown in Table 3. Patients with AKI-2 and AKI-3 were more likely to have category global(G) lesions than No AKI patients; the proportion of patients with type IV + V was significantly lower in group AKI-2 and AKI-3 compared with group No AKI; the proportion of patients with the active plus chronic lesions and great crescents (involves > 50% of the circumference of Bowman’s capsule) ≥50% was significantly higher in group AKI-3 than the other three groups.

**Table 3:** The histological features on the basis of ISN/RPS

Parameter	no AKI	AKI-1	AKI-2	AKI-3	*P*
n(%)	82 (49)	40 (24)	26 (16)	19 (11)	
ISN/RPS LN IV + V	30 (37)	14 (35)	5 (19)	1 (5)	0.03
ISN/RPS IV-G(n[%])	61 (74)	33 (83)	24 (92)	18 (95)	0.07
ISN/RPS IV-A + C(n[%])	12 (15)	15 (38)	6 (23)	9 (47)	< 0.01
Great crescents					
< 30%(n[%])	69 (84)	33 (83)	19 (73)	5 (26)	
≥30,< 50%(n[%])	10 (12)	4 (10)	3 (12)	3 (16)	
≥50%(n[%])	3 (4)	3 (7)	4 (15)	11 (58)	< 0.01

*ISN/RPS* International Society of Nephrology and Renal Pathology Society, *IV-G* diffuse global lesions; Great crescent is one of those extracapillary lesions that involves > 50% of the circumference of Bowman’s capsule; great crescents ≥50%, refers to patients with > 50% of the glomeruli containing great crescents

### Primary endpoint outcomes

**Fig. 1:**
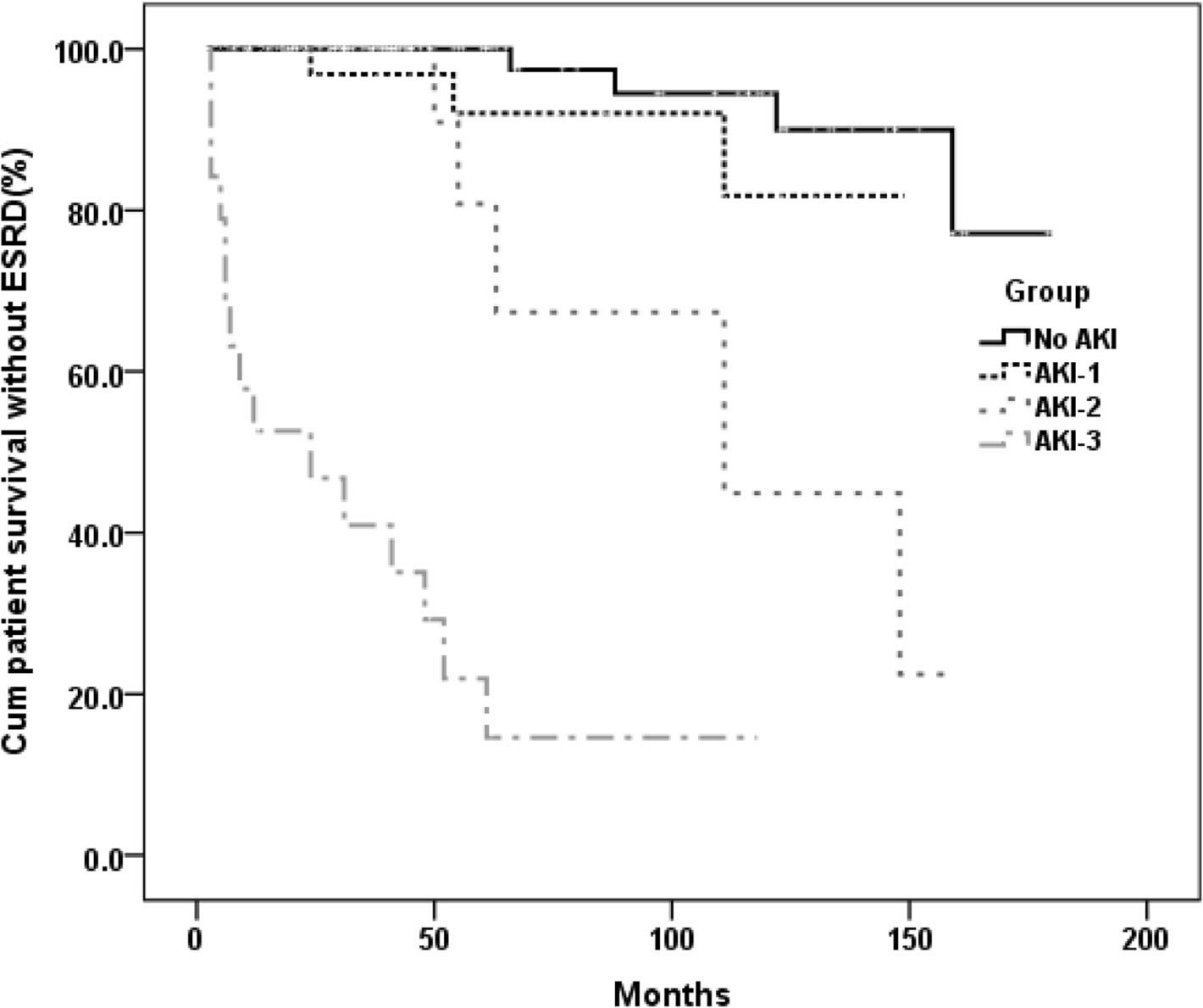
Patient survival without ESRD in DPLN patients based on AKI stage (*P* < 0.001, overal). AKI-1 versus No AKI: 137 ± 7 mon vs 169 ± 5 mon,*p* = 0.209; AKI-2 versus No AKI:112 ± 15 mon vs 169 ± 5 mon, P < 0.001; AKI-3 versus No AKI: 37 ± 9 mon vs 169 ± 5 mon *P* < 0.001. AKI-2 versus AKI-1: 112 ± 15 mon vs 137 ± 7 mon *p* = 0.089; AKI-3 versus AKI-1: 37 ± 9 mon vs 137 ± 7 mon, *P* < 0.001. AKI-3 versus AKI-2: 37 ± 9 mon vs 112 ± 15 mon, *P* < 0.001

The mean follow-up of all patients was 5.1 ± 3.8 years. The patient survival without ESRD of all patients was 86% at 5 years and 79% at 10 years. The incidence of ESRD and death increased with advancing KDIGO AKI stage. The patient survival rate without ESRD at 10 yr was 94.5% for No AKI patients, 81.8% for AKI-1 patients, 44.9% for AKI-2 patients and 14.6% for AKI-3 patients. Patient survival without ESRD was significantly worse for patients with AKI-2 (mean, 112 ± 15mon) and AKI-3 (mean, 37 ± 9mon) compared with No AKI (mean, 169 ± 5mon,*P* < 0.001);Patient survival without ESRD was more likely to have short survival time for patients with AKI-1(mean, 137 ± 7mon,) than No AKI (mean, 169 ± 5mon,*P* = 0.08).(Fig. 1).

**Fig. 2:**
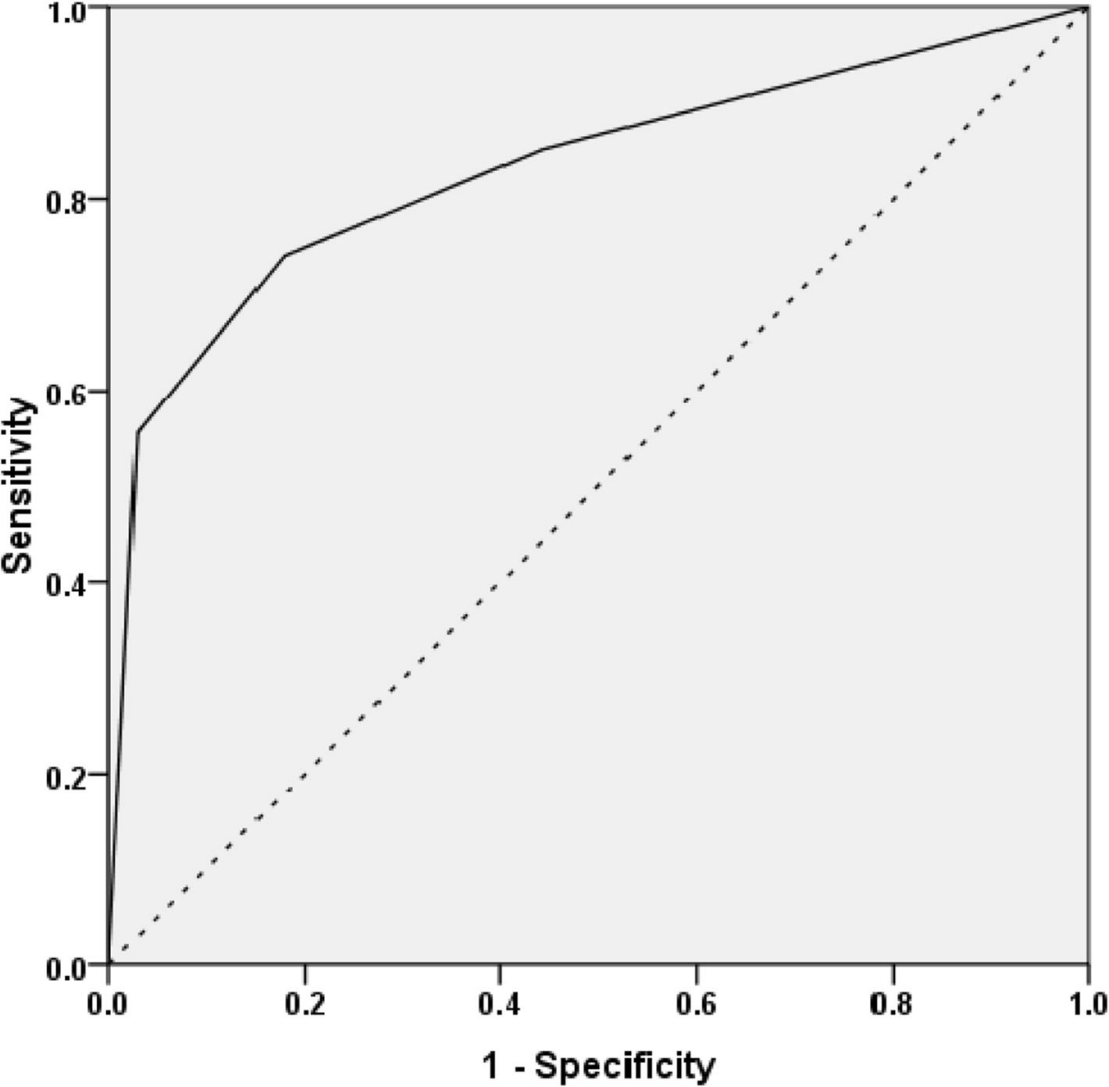
ROC curves for AKI classification to predict primary outcome (AUROC curve is 0.831, *P* < 0.001). The sensitivity and specifity at the cut-off level of AKI-2 was 74 and 82%

The ROC curve model represents the true-positive and false-positive rates for progression to primary end point and the area under the ROC curve for progression to primary end point was 0.83 (95% CI: 0.73–0.93) (*P* < 0.001) (Fig. 2). AKI-2 stage was the best cut-offs for clinical use and had higher sensitivity or better ability to identify DPLN patients with primary outcome(the sensitivity was 74% and specificity was 82%).

There was a significant dose-dependent relationship between the severity of AKI and the primary endpoint. In Cox regression analysis (Table 4), AKI stage was independently associated with primary endpoint, with an adjusted HR of 3.8(95% CI 2.1–6.7, *P* < 0.001). Great crescent > 30% (HR = 2.1, 95% CI 1.2–3.6, *P* < 0.008), Hb < 8.0 g/dl (HR = 4.8, 95% CI 1.6–14.0, *P* < 0.005) and proteinuria> 3.0 g/d (HR = 5.1, 95% CI 1.5–17.1, *P* < 0.008) were also the main predictors of ESRD.

**Table 4:** Cox regression analyses-adjusted HR for for primary endpoint

variables	HR	95%confidence intervals	*P-*value
AKI stage	3.8	2.1–6.7	< 0.001
C_3_ > 0.4 mg/dl	0.7	0.2–2.1	0.49
MP pulse treatment	0.8	0.2–2.5	0.68
INS/PRN LN type IV + V	0.9	0.7–1.2	0.44
INS/PRN active+chonic lesion	1.1	0.3–3.5	0.89
great crescent> 30%	2.1	1.2–3.6	0.008
mBP > 115 mmHg	2.6	0.9–7.7	0.081
Hb < 8.0 g/dl;mean ± SD)	4.8	1.6–14.0	0.005
Proteinuria> 3.0 g/d	5.1	1.5–17.1	0.008

*AKI* acute kidney injury, *C*_*3*_ serum complement component 3, *MP* methylprednisolone, *LN* lupus nephritis; great crescent, is one of those extracapillary lesions that involves > 50% of the circumference of Bowman’s capsule; great crescents ≥30%, refers to patients with > 30% of the glomeruli containing great crescents; *mBP* mean blood presure, *Hb* hemoglobin

## Discussion

There are more options for treatments in recent years, but the efficacy does not appear to be improved obviously in DPLN patients. The patient and kidney 10-year survival rate of DPLN patients was 79% in 1997and 82.8%in 2006 [16, 17]. During a mean follow-up of 5.1 ± 3.8 years in our population, the patient survival without ESRD of all DPLN patients was 79% at 10 years. Current treatment approach for LN, as outlined in the recommendations by international medical associations including European League Against Rheumatism (EULAR), the ACR or KDIGO, still was corticosteroids in combination with CTX or MMF as induction treatment and azathioprine or MMF as maintenance treatment [18–20]. No consensus has been reached on the role of calcineurin inhibitors (CNIs) and rituximab so far. The dose of corticosteroids and cytotoxic agents is mostly eminence-based rather than evidence-based. The initial dose of prednisone in proliferative LN was different from 0.5 to1.0 mg/kg per day, preceded by three iv. pulses of MP (the dose from 6.6 mg/kg per day to 1.0 g/day) in some clinical trials [21–26]. MP pulse regimen was preferred for severe clinical conditions of proliferative LN by nephrologists. In our study, 68% patients in group AKI-3 and 62% in group AKI-2 had MP pulse therapy, only 24% in group No AKI.

The patient survival rate without ESRD at 10 yr was 94.5% in No AKI patients, which was similar with the result of other reports [3, 27, 28]. More than 60% DPLN patients with AKI-2 and AKI-3 stage received intravenous MP pulse therapy, but the renal survival rate can’t be improved effectively. The patient survival rate without ESRD at 10 yr was 44.9% for AKI-2 patients and only 14.6% for AKI-3 patients. DPLN with AKI had poor short-term renal outcome in our previous study. Now we demonstrate AKI is a serious complication of DPLN with adverse long-term outcomes. Cox regression analyses showed a relationship between the increased severity of AKI and increased incidence of ESRD.

Systematic review with meta-analysis and recent observational studies demonstrated a reproducible association between AKI, subsequent CKD and ESRD [29–33]. The populations of these studies came from cardiovascular diseases, ICU, general surgical settings and general hospital settings and the causes of AKI in these patients usually were renal hypoperfusion, acute tubular injury or necrosis, renal artery stenosis and nephrotoxic drugs (such as contrast agents). Our study showed the association between AKI and ESRD in glomerulonephritis patients. Furthermore, the ROC curve confirmed the good discriminatory power of the KDIGO AKI classification in predicting long-term outcome of DPLN with AKI. Such analytical results (AUROC was 0.831,*P* < 0.001) suggest that AKI classification is a good tool for measuring disease severity in lupus patients with AKI.

AKI is a heterogeneous syndrome with multiple potential causes and the outcomes may differ across different AKI etiologies, severity of primary disease and complications. Clinical settings of proteinuria and pathological changes will have an impact on outcomes in DPLN with AKI patients. Relative risks were particularly high in DPLN with AKI patients who had heavy proteinuria (24-h urine protein> 3.0 g, HR = 5.1). Besides, the multivariate Cox regression analysis revealed that anemia (Hb < 8.0 g/dl, HR = 4.8) and crescents (more than 30%, HR = 2.1) were independent risk factors for ESRD. These risk factors were also reported in recent studies from Asian LN patients [34–36].

The most important limitation of this study was the study design. It was a retrospective observational study which is commonly affected by various sources of bias. It should be stressed, however, that the primary outcome (ESRD and death) was the hard endpoint and therefore unlikely to have been influenced by knowledge of patients’ allocation. This study was not a clinical trial to evaluate therapeutic effect of drug intervention, so our doctors were not biased against certain groups of patients during follow-up. Second, this study was a relatively small size with Chinese patients rather than a large international multicenter study. Our small size study may over-estimated the magnitude of an association between risk factors and renal outcomes. The required sample size was 122 patients based on statistic power of a test (80%) and type I error (5%), so 167 patients in our study were sufficient to prognostic analysis of renal outcome using Cox regression model.

## Conclusions

We found that each increase in severity of KDIGO AKI category was associated with an increase in progression to ESRD in DPLN patients. Analytical data also confirmed the good discriminative power of the KDIGO AKI classification system for predicting long-term prognosis of DPLN patients with AKI.

## Data Availability

The data that support the findings of this study were used under license for the current study from the First Affiliated Hospital of Wenzhou Medical University and are not publicly available. Data are, however, available from the authors upon reasonable request and with permission of the First Affiliated Hospital of Wenzhou Medical University.

## Abbreviations

AKI: Acute kidney injury
DPLN: Diffuse proliferative lupus nephritis
eGFR: Evaluated glomerular filtrition rate
ISN/RPS: International society of nephrology/renal pathology society
KDIGO: Kidney Disease: Improving Global Outcomes
LN: Lupus nephritis
SLE: Systemic lupus erythematosus
